# Phycotoxicity and catalytic reduction activity of green synthesized *Oscillatoria* gelatin-capped silver nanoparticles

**DOI:** 10.1038/s41598-022-22976-6

**Published:** 2022-11-27

**Authors:** Rasha A. Abo-Elmagd, Ragaa A. Hamouda, Mervat H. Hussein

**Affiliations:** 1 Botany department, Faculty of Science, Mansoura University, Mansoura, Egypt; 2 Department of Microbial Biotechnology, Genetic Engineering and Biotechnology Research Institute, University of Sadat City, Sadat City, Egypt; 3 Department of Biology, College of Sciences and Arts Khulais, University of Jeddah, Jeddah, 21959 Saudi Arabia

**Keywords:** Biological techniques, Biotechnology, Microbiology

## Abstract

Over the last decade, an extensive range of consumer products containing manufactured silver nanoparticles (AgNPs) have been progressively used. The unfitting usage and discharge of these materials can enable passage of AgNPs into the aquatic ecosystem causing prospective toxicological consequence. The present study shed new lights on the phycotoxicity of small (8.47–17.66 nm) and stable *Oscillatoria* reduced gelatin-capped silver nanoparticles (OG-AgNPs) fabricated using a completely green synthetic technique. In this work, estimating of the possible toxic effects of OG-AgNPs on two freshwater microalgae *Chlorella vulgaris* and *Chlorella minutissima* was carried. This study found that, the growth of cells and photosynthetic pigment inhibitory effects of OG-AgNPs exhibit a significant increase with increasing time and concentration compared to control. Based on the IC_50_ value *C. vulgaris* (3.705 μg/mL) was found to be more sensitive to OG-AgNPs than *C. minutissima* (5.8 μg/mL). This study revealed that OG-AgNPs exhibit potent phycotoxic effect against *Chlorella* species. Finally, the negative effect of OG-AgNPs on aquatic algae and these modifications might have severe effects on structure and function of aquatic ecosystems. Besides, the biosynthesized OG-AgNPs showed a catalytic activity in the reduction of hydrogen peroxide, one of the reactive oxygen species that represent a major threat to biological systems. This method pretends an auspicious non-skill dependent technique with a good sensitivity for determination of H_2_O_2_ concentration, particularly at trace ppm level for applying in numerous domains such as medical and industrial processes.

## Introduction

Metal particles at the nanoscale have attracted the attention of scientists and engineers owing to their dramatic influences and unusual manners that modify a variety of characteristics, including biological and physico-chemical properties^1,2^. The development and current headway of nanoscience and nanotechnology initiate new prospects for the application of nanomaterials in numerous fields. Among the metallic nanoparticles (MNPs), silver nanoparticles (AgNPs) are believed to be one of the most worthy nanoparticles because of their distinctive properties, capability to compose various nanostructures and low cost of production^3,4^. Silver nanoparticles possess unique properties and enormous medications for various applications such as bio-sensing, catalysis, cancer treatment, agriculture and wastewater treatment, healthcare, drug delivery, and industrial and antimicrobial applications due to the high surface to volume ratios permitting great discernibility in the fluid form^5–9^. Metal nanoparticles prepared in polymer matrices are more stable^10,11^ and have potential applications in nanoelectronic devices, sensors, molecular optical devices, and engineering nanocomposites with well-defined properties^12–15^. Nowadays, green synthesis methods have more benefit than over other classical synthesis techniques due to the accessibility of more biological materials and eco-friendly routes. The green synthesis of eco-friendly nanoparticles from algae and its application in the medicinal sector are respected as building blocks to control many diseases^16,17^. For completely green fabrication of silver nanoparticles, several biopolymers such as gelatin, starch, chitin, glucose, cellulose, and polyvinyl alcohol (PVA) have utilized as reducing and capping agents^18–21^. Currently, the expansion in the utilization of AgNPs-containing products such as dressings, socks, air filters, refrigerators, healthcare, and cosmetic products, washing machines, electronics, and medical devices like catheters, implant surfaces, wound dressings, and plasters^21^ can cause discharging of metallic AgNPs and Ag^+^ cations into eutrophic freshwater, brackish and marine ecosystems^22^. Consequently, ecological worries have been increased worldwide as a result of this elevation in consumption and the emission of AgNPs into the environment as a major ecological and potential health problem^23^. Furthermore, silver metal is considered the second most deadly metal to aquatic organisms after mercury^24^. Blaser et al*.*^25^ reported that the presence of silver nanoparticles in water may have a high ability to move freely and can be easily moved to the huge aquatic environment, But, their ecological influence on aquatic environments is still indefinite. Boyle^26^ reported that the response of algae species to diverse toxic chemicals differ broadly. Several studies have been executed to investigate the toxicity of silver nanoparticles on many species of marine and freshwater phytoplankton, including *Chlorella vulgaris* and *Dunaliella tertiolecta*^27^, *P. subcapitata*^28^, cyanobacteria^29^, *M. aeruginosa*^30^, red marine algae *Corallina elongata* and *Gelidium amansii*^31^. Reactive oxygen species (ROS) such as hydrogen peroxide, hydroxyl radical, and superoxide have been depicted as potential contaminants in both the aquatic environment and biological systems. These chemical substances are products of oxygen metabolism in aerobic organisms. DNA has an essential role in preserving the life activities of living organisms^32^. Hydrogen peroxide (H_2_O_2_), is one of the reactive oxygen species, when present in excess, it damages DNA in living systems^33^. Consequently, the detection of H_2_O_2_ in trace concentrations has become an important and required task. It is also important in a variety of fields, including industry, medicine, and the environment^34,35^. Currently, many approaches have been applied for the determination of H_2_O_2_ concentration and give high sensitivity^36,37^. However, these procedures have some downsides as they are not cost-effective and include time-consuming schemes that are skill-dependent. Interestingly, that are utilization of nanoparticles as colorimetric sensors has been highlighted as a method for following environmental toxins in biological systems that presents simple and cost-effective procedures with excellent sensitivity. Numerous studies have shown that Ag-nanocomposite reveals a catalytic activity for the decomposition and detection of H_2_O_2_^2,38,39^. The development of a green method for the synthesis of AgNPs gained great attention. Because it is offers headway to the development of eco-friendly, low-cost, single-step, non-toxic, and biocompatible techniques for the synthesis of nanoparticles. The aim of this study is to determine the ability of *O. limnetica* to produce small, highly stable well dispersed gelatin-capped silver nanoparticles (OG-AgNPs) with anisotropic nanostructures via a completely green synthesis approach, and investigate the toxicity of OG-AgNPs by using two freshwater microalga *C. vulgaris* and *C. minutissima.* It is possible using biofabricated OG-AgNPs as optical sensor for determination of H_2_O_2_.

## Materials and methods

### The cyanobacterium and culture conditions

*Oscillatoria limnetica* was obtained from the algal culture collection of the Phycology Lab at faculty of science, Mansoura University, Mansoura, Egypt. *Oscillatoria limnetica* was inoculated into 500 ml Erlenmeyer flasks containing 300 ml nutrient medium (BG11) using the batch culturing system. Cultures were incubated under continuous illumination (57.75 µmol photons m^−2^ s^−1^) at 28 ± 1 °C for 21 days representing the onset of the stationary phase. *O. limnetica* aqueous extract was prepared after Hamouda et al.^5^ and stored at 4 °C.

### Green synthesis gelatin-coated silver nanoparticles

Gelatin coated silver nanoparticles was green synthesized after Mohan et al.^11^ using the reducing potential of *O. limnetica* extract. In 250 ml Erlenmeyer flask, 0.1 g gelatin was mixed with 90 mL distilled water and exposed to heating at 40 °C till a clear solution was obtained. Then, this solution was supplemented with 5 mL of AgNO_3_ solution (0.5 M) with continuous stirring to obtain Ag^**+**^/gelatin solution. After that, 10 ml of cyanobacterial extract was added under continuous stirring at 38 °C in light (57.75 µmol m^−2^ s^−1^) and let the reaction to proceed for 4 h. For investigating particles growth, aliquots were collected at the following time intervals (1, 2 and 4 h.). A change in the color of the solution from light green to dark brown designated the formation of *Oscillatoria* gelatin-capped silver nanoparticles (OG-AgNPs). A UV–vis spectro-photometer (ATI Unicam 5625 UV/VIS Vision Software V3.20) was used to accomplish absorption spectra in the wavelength range 200–900 nm. Finally, the solution was centrifuged at 4528×*g* for 20 min using MIKRO 12 Hettich Zentrifugen D-78532 centrifuge (Tuttlingen-Germany). Then, the OG-AgNPs were washed by deionized water for 4–5 times, dried and stored at 4 °C till further characterization and application.

### Characterization of OG-AgNPs

#### UV–visible and FT‑IR spectral analysis

Production of OG-AgNPs was approved by ultraviolet–visible spectral analysis of the nano solution in the region of 200–900 nm using scan UV–Vis spectrophotometer. Samples (200 μl aliquot of biosynthesized OG-AgNPs solution (2.5 μg/ml OG-AgNPs concentration) were stationed in a quartz cuvettes for measurement.

FTIR spectroscopy measurements of the lyophilized OG-AgNPs were performed by ThermoFisher Nicolete IS10, 098e3wq (USA) in the wave number range of 4000–500 cm^−1^ at a resolution of 1 cm^−1^ using potassium bromide (1: 100).

#### Transmission electron microscopy (TEM)

Particles size, morphology and crystallography of the biofabricated gelatin capped-AgNPs were distinguished by transmission electron microscopy (JEOL, JEM-2100, Japan) at the Electron Microscope Unit, Mansoura University. TEM worked at an accelerating voltage of 200 kV. Drops of aqueous solution of silver nanoparticles were put on carbon-coated copper grid and dried under infrared lamp former to analysis.

#### Energy-dispersive X-ray spectroscopy (EDX) study

Elementary analysis of the produced OG-AgNPs was estimated by the energy-dispersive X-ray spectroscopy (EDX) by a field-emission of scanning electron microscope using “Oxford X-Max 20 Instrument at the Electron Microscope Unit, Mansoura University, Egypt.

#### X-ray diffraction analysis (XRD)

The X-ray diffraction analysis was maintained using X-ray diffractometer (Philips X’pert Pro, Panalytical) to study the crystalline structure of OG-AgNPs operating with CuKa (k = 1.540 A°) radiation and a programmable divergence slit (40 kV, 20 mA). The diffracted intensities were documented at 4–80 °C (2θ). The crystallite domain size was calculated using the Debye–Scherrer formula at Central Metallurgical R&D Institute, Cairo-Egypt.

#### Phytochemical screening of *O. limnetica* extracts

Phytochemical detection of Quinines, Saponins, Flavonoids and Terpenoids was executed for aqueous extract of *O. limnetica* as per standard procedures illustrated by Sanjeet et al*.*^40^ and Subathraa et al.^41^.

#### Phycotoxicity assay

Toxicity evaluation of the biosynthesized *Oscillatoria* gelatin-capped silver nanoparticles (OG-AgNPs) was investigated on two freshwater microalgae *C. vulgaris* and *C. minutissima* were computed according to the standard commands of microalgae growth inhibition test in accordance to OECD (No. 201). *Chlorella vulgaris* and *C. minutissima* were obtained from algal Collection, Phycology Lab, faculty of Science Mansoura University as test algae with cell density ranged 10^4^–10^5^ cells mL^−1^ at the late exponential growth phase (5 days) to test the growth inhibition. In this experiment, the two *Chlorella* species were exposed to eight gradually increasing nominal concentrations of freshly prepared OG-AgNPs solution including a control (100, 50, 25, 12.5, 6.5, 3.123, 1.5 and 0 μg/mL), as 1 ml of each concentration were added to Erlenmeyer flasks containing 30 ml of BG-11 culture media. Then, flasks were inoculated with 5 ml of *Chlorella* sp. at a final cell density of (119 cells/mL) for *C. minutissima.* and (76 cells/mL) for *C. vulgaris*. All experiments were performed in triplicate and were incubated for 4 days at 28 ± 1 °C under continuous light condition (57.75 µmol m^−2^ s^−1^). For growth evaluation and physiological parameters including photosynthetic pigments (chlorophyll *a*, chlorophyll *b* and carotenoids) contents, 100 μl aliquots of the micro algal cultures were sampled each 24 h. intervals in triplicate and the growth inhibition test were performed according to OECD guidelines. Growth was determined by manual-cell counting using standard haemocytometer under light microscope. Growth inhibition was elucidated as cell yield at the end of the incubation period, cell count along the incubation period (24 h. interval) for each treatment. Besides, the toxicity of OG-AgNPs was presented as percent microalgal growth inhibition and calculated as following:

$${\text{Green algae Inhibition efficiency}}\% = \left[ {\frac{{{\text{A}} - {\text{B}}}}{{\text{A}}}} \right] \times 100$$

where A is the cell count/mL of the control culture after 96 h of incubation period. B is the cell count/mL of the different concentrations of the test treatments after 96 h of incubation periods.

The average specific growth rate which is determined as the logarithmic increase in cell count was calculated after 96 h. using following equation:

$$\mu i - j = \frac{\ln Xj - \ln Xi}{{tj - ti}}\left( { day - 1} \right)$$

As, µ_i-j_ is the average specific growth rate from time i to j; t_i_ is the initial time of exposure period, t_j_ is the final time of exposure, X_i_ is the cell count at time i, and X_j_ is the cell count at time j.

Additionally, the percent inhibition of growth was estimated via the following equation:

$$I_{r} \% = \left[ {\frac{{\left( {\mu c - \mu t} \right)}}{{\mu c}}} \right] \times ~100$$

where Ir % is the percent inhibition of the average specific growth rate, μc is the mean value of the average specific growth rate in the control, and μt is the average specific growth rate after treatment.

The 50% effective concentration (EC_50_) of samples was designated as the concentration corresponding to the point where I is 50% on the inhibition curve.

Photosynthetic pigments were extracted in 85% acetone and Chlorophyll *a*, chlorophyll *b* and carotenoids were evaluated for each sample by measuring the optical density using UNICO UV-2000 spectrophotomer at wavelengths 470, 645 and 662 nm as explained by Metzner et al*.*^42^.

### Investigation of catalytic effect of H_2_O_2_ on the synthesized gelatin coated silver nanoparticles

The experimental method for evaluation of the sensing character of *Oscillatoria* gelatin-capped AgNPs (OG-AgNPs) was assessed according to Endo et al*.*^43^ For estimation of the optical characteristics of OG-capped AgNPs solution as a spontaneous peroxide sensor, hydrogen peroxide solutions (1000 μl) with various concentrations (1–10% H_2_O_2_) were mixed with the silver nanoparticles solution (catalyst) in a quartz cuvette at a ratio of 1: 1.5. To detect the H_2_O_2_, the gradient of color change and the plasmon resonance surface (SPR) shift were investigated. As, the UV–vis spectrum variation in response to H_2_O_2_ different concentrations as a result of the catalytic reaction between silver nanoparticles and hydrogen peroxide was screened at room temperature using UV–Vis spectrophotometer between 300 and 600 nm after 2 min of incubation.

#### Statistical analysis

All data of this study were expressed as means ± SD. The statistical analysis was conducted using SPSS software version (Version, 19, IBM SPSS, Armonk, NY, USA).

## Results and discussion

### Optical characteristics of the biosynthesized *Oscillatoria* gelatin-coated Ag-NPs

**Figure 1:**
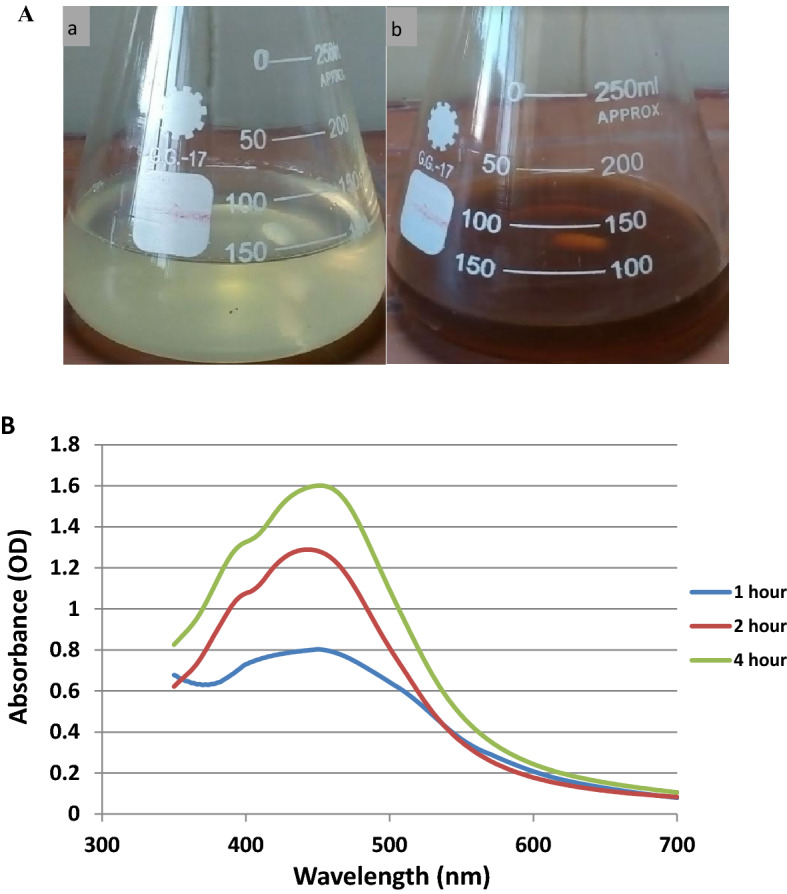
(**A**) aqueous solution of *Oscillatoria limnetica* **(a)** and biosynthesized *Oscillatoria* gelatin-capped AgNPs colloid **(b)** after an ahour of reaction, (**B**) Absorption spectra of the biosynthesized *Oscillatoria* gelatin-capped AgNPs.

Currently, bio-nanotechnology is an eco-friendly technology for the fabrication of nanoparticles. In this study, *O. limnetica* has attested to be a significant biological partner in the green synthesis of stable AgNPs^5^. Herein, gelatin was provided as both stabilizing and capping agent owing to its well biodegradability and biocompatibility whereas, the amine postponed groups on the gelatin backbone stabilize and prevent aggregation of AgNPs^44^. During this reaction, the color of Ag^+^/gelatin solutions converted from greenish to light brown and then dark brown by prolonging the reaction time that indicating the synthesis of *Oscillatoria* reduced gelatin-capped Ag-NPs (OG-AgNPs) of various particle sizes (Fig. 1A)^45^. The color transition was assigned to the excitation of surface plasmon absorption in the synthesized OG-AgNPs solution as mentioned by Forough et al.^46^. Confirmation and characterization of OG-AgNPs at different reaction times was detected by UV–Vis spectroscopy, which was acted as a preliminary tool for recognizing AgNPs in aqueous solutions. Figure 1B; illustrates UV–vis spectra of biosynthesized OG-AgNPs at different reaction intervals which exposed characteristic absorption bands in the range of 400–500 nm due to the surface plasmon resonance (SPR) of OG-AgNPs^6,47^. Spectral data demonstrates the absorption maximum peaks of the OG-Ag-NPs that were slightly red-shifted from 446 to 449 nm with increasing intensity (0.8–1.6 nm) by increasing the reaction time implying that, the nanoparticle size increased with increasing reaction time. Consequently, this rise in the SPR intensity of the peak as a function of reaction time designates continual reduction of cations (Ag^+^) followed by increasing production of the Ag-NPs^5^. The present results were in accordance with that of Govindaraju et al*.* and Jena et al.^48,49^ who initiated their discussion on broadening of the SPR peak width is respected an agreeable detector of the size of nanoparticles and its Polydispersity, where the range of 320–580 nm is characteristic λmax for AgNPs biosynthesis. Moreover, Sharma et al*.*^50^ suggested that frequency and band width of SPR is not only depending on both size and shape of the nanoparticles, but also on the dielectric constant characterized the metal itself as well as adjacent medium. Consequently, shifting in the position of spectral peaks may be resulted in increasing broadening of peaks owing to increase in particles size. The current results are in accordance with the findings of Lowry et al*.*^51^ who elucidated that size, number and shape of the manufactured NPs are dependent on concentration and time of exposure to silver ions carried out using *Spirulina platensis.*

### Capping

Capping of metal nanoparticles is one of the significant issues for its stability, although produced atoms considered as a nucleation center due to its surface activity that catalyze gathering of metal ions and forming large metal clusters which mean an extension in reduction process^52,53^. Metal nanoparticles (MNPs) aggregation can be prevented by capping agents that offer good dispersion of nano particles, additionally capping agents may prevent metal nanoparticles from reacting with oxygen therefore preventing oxidation. Morsy et al. and Pooja et al*.*^53,54^ stated that metal nanoparticles could be physically stabilized in the presence of capping agents such as a natural polymer which adsorbed to nanoparticles surface and caused a steric repulsion between particles owing to polymer moieties similar charge. The current study demonstrated the complete coating of AgNPs by gelatin as observed (Fig. 3). Gelatin could be functioned as stabilizing agents in the biosynthesis of AgNPs as positively charged gelatin can be electrostatically adsorbed on negatively charged AgNPs to form gelatin-coated AgNPs. Presence of protein in the reduction solution of *O. limnetica* acts as a capping agent and provides additional stability to AgNPs as well as gelatin exhibited well-capping activity^55^.

**Figure 2:**
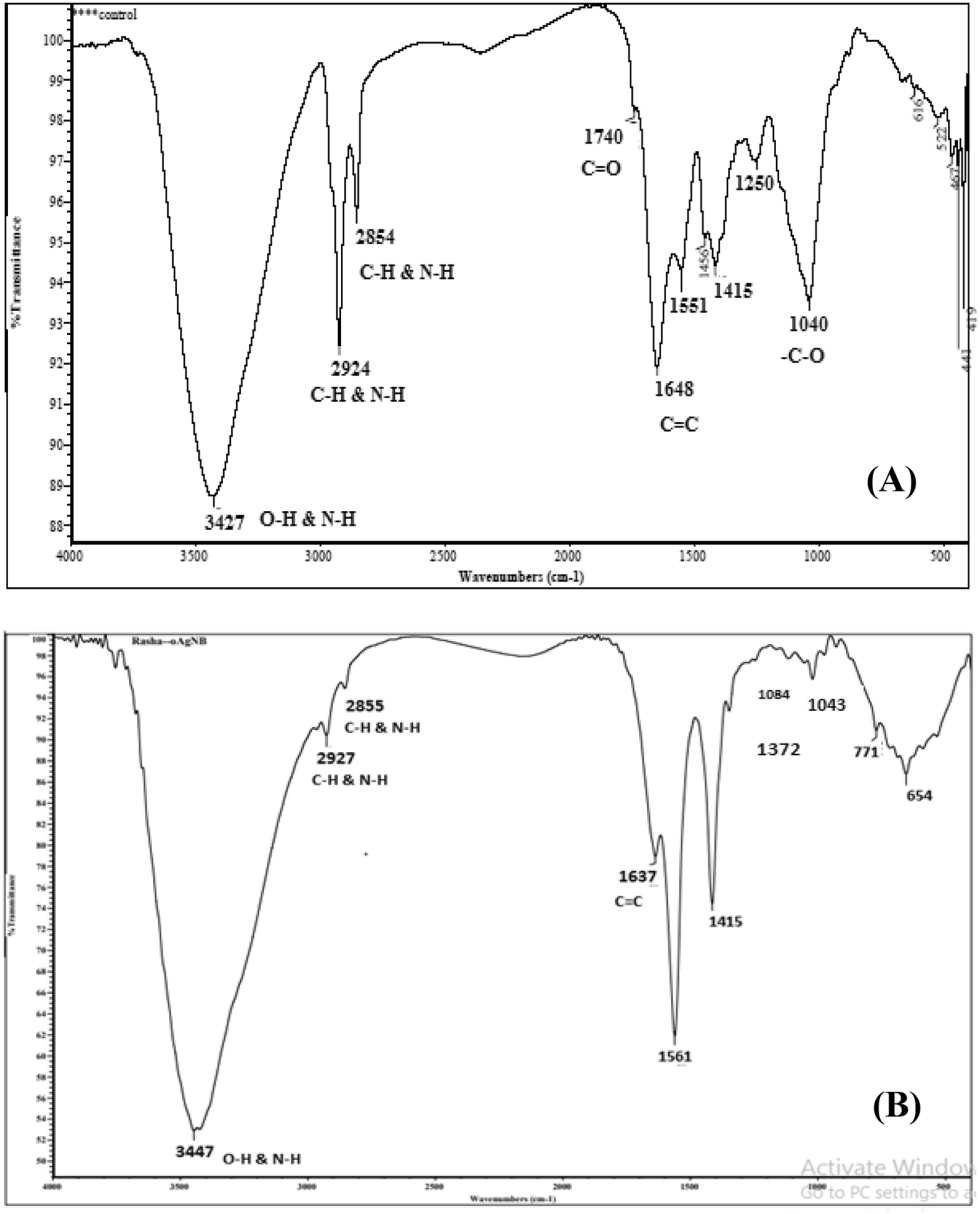
FT-IR spectrum verified by making KBr disc with *O. limnetica* extract (**A**) and biosynthesized *Oscillatoria*- gelatin capped silver nanoparticles (**B**).

**Figure 3:**
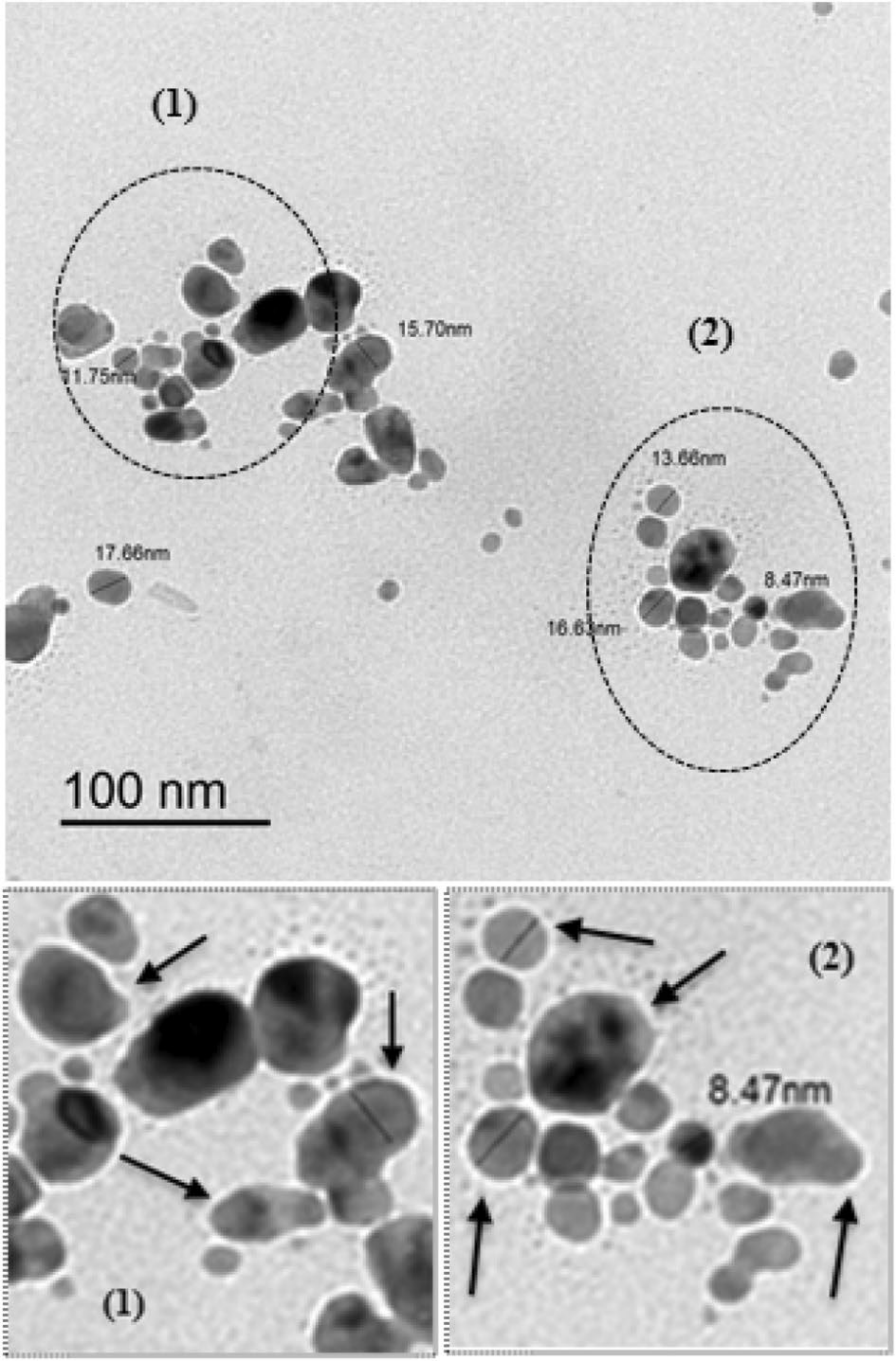
TEM image of fabricated *Oscillatoria* gelatin capped silver nanoparticles at 1 h with size in the range 8.47–17.66 nm.

### FTIR spectroscopy of OG-AgNPs

FTIR spectral analysis was used to study the biomolecules, which were involved in the reducing and formation of *Oscillatoria* reduced gelatin capped silver nanoparticles and to ascertain the capping by gelatin^56^. The FTIR spectra of OG-AgNPs biosynthesized by *O. limnetica* and *O. limnetica* extract was illustrated in Fig. 2A,B. FTIR profile (Fig. 2A) demonstrated 14 peaks posts at 3427, 2924, 2854, 1740, 1648, 1551, 1455, 1415, 1250, 1040, 616, 522, 467 & 441 cm^−1^ for *O. limnetica* extract as mentioned by *Hamouda *et al.^5^. Whereas, the spectra of biosynthesized OG-AgNPs revealed 11characteristic absorption bands at 3447, 2927, 2855, 1637, 1561, 1415, 1372, 1084, 1043, 771 & 654 cm^−1^ (Fig. 2B).

The distinctive broad sharp peak at 3427, 3447 cm^−1^ may be corresponded to O–H functional groups in phenols, flavonoids, and N–H stretching vibration of primary and secondary amines of amino acids and proteins^5,57,58^. This spectral pattern ascertained the reducing action of *Oscillatoria limnetica* extract, as the shifting in AgNPs spectral profile may be assigned to the interactions between those chemical functional groups and silver nanoparticles^59^. Cooperation between protein molecules and AgNPs by free amide was recorded^60,61^. FTIR spectroscopy data demonstrated that the amide linkage of the protein held the higher potential to join silver and subsequently creating protein covering around AgNPs to protecting it from agglomeration and thus stabilize the medium^62^. The bands at 2927, 2854 and 2924, cm^−1^ pointed to C–H group representing stretching vibration of alkane and N–H bending vibration^57^. There was a characteristic peaks at 1648, 1637, 1551 and 1561 cm^–1^ which could be attributed to amides (N–H) stretching of peptide linkage of protein^64^ and C=C stretching which may implicate in stabilizing nanoparticles by proteins as described by Castro et al*.*^63^. A significant peak at 1740 cm^−1^ can be assigned to aldehydic group (C=O). Furthermore, peaks designated at 1040 and 1250 cm^−1^ may be assigned to either sulfur or phosphorus function groups, which maybe attach silver and proceed both capping and stabilizing process of nanoparticles^63^. On the other hand, the small peak at 1043 cm^−1^ could be corresponded to the –C–O vibrations from the gelatin^64^. Meanwhile, the two minor peaks at 1084 cm^−1^ and 1372 cm^−1^ assigned to the –C–O stretching and O–H stretching. A small absorption band at 1415 cm^−1^ may be corresponded to N–H, the amide linkage vibration of the gelatin. Additionally, the peak observed at 771 and 654 cm^−1^ could be ascribed to the bending area of the aliphatic chain. This analysis confirms the capping of Ag-NPs by gelatin as there was a small shift in the O–H and N–H bands of the gelatin-capped Ag-NPs compared to free gelatin implied that, there was electrostatic crosslinking between the silver nanoparticles and gelatin^65^.

### TEM analysis

The morphology and sizes of the bio synthesized OG-AgNPs were detected using Transmission electron microscopy (TEM). TEM micrograph (Fig. 3) depicted that synthesized silver nanoparticles are small, well dispersed and quasi-spherical in shape with anisotropic nanostructures. In addition, the TEM results demonstrated that the particles size ranged between 8.47 and 17.66 nm (2 h reaction time) having good distribution with no clusters^66^.

### EDX analysis

**Figure 4:**
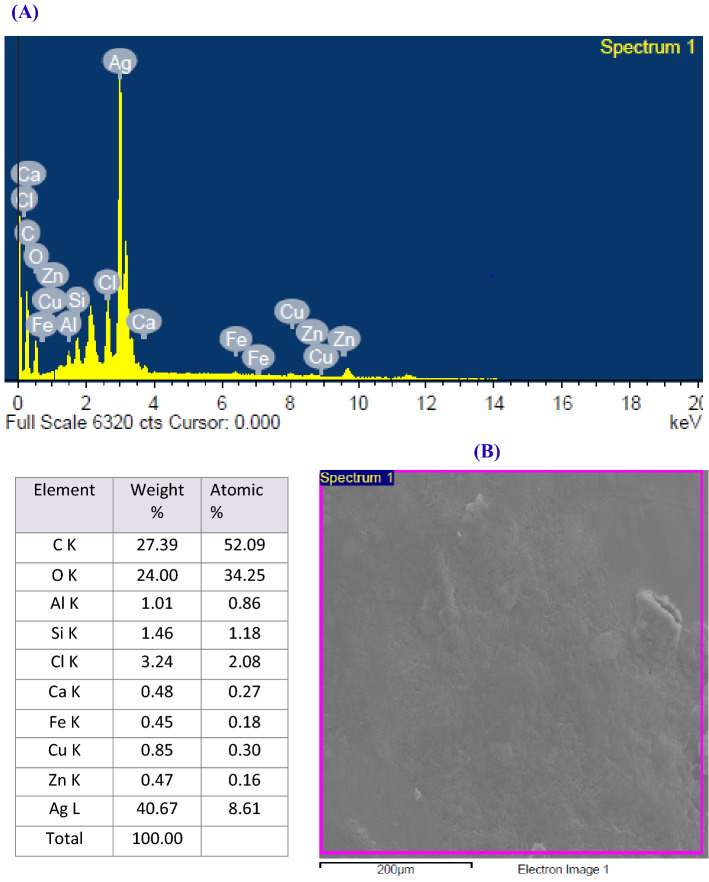
Energy-dispersive X-ray (EDX) spectrum of the biosynthesized OG-AgNPs recorded revealing peak approximately near 3 keV confirming the presence of silver (**A** and **B**).

EDX and mapping spectrum were carried out to investigate the elemental composition of the biosynthesized nanoparticles and capping molecules. In this study, EDX spectrum proved the significant existence of 40.67% silver (Fig. 4) and a sharp signal was elucidated at approximately 3 keV which confirmed the presence of elementary silver in the nano-scale and could be attributed to the strong surface plasmon resonance (SPR)^67^. Other additional peaks for C, O, Zn, Cu, Fe and Cl were also detected in the EDX spectrum owing to the protein and biomolecules capping the synthesized OG-AgNPs^68^ that were leaked out from *O. limnetica*. This result is in line with the result of Ali et al.^69^, who studied the biosynthesis and characterization of silver nanoparticles using the marine cyanobacterium, *Oscillatoria wellei* NTDM01, where they found that EDX analysis exhibit the biofabriction of AgNPs in high percentage with traces of magnesium, calcium and chloride. In addition, the presence of aluminum is because of aluminum grid sample holder used to support the sample.

### X-ray diffraction

**Figure 5:**
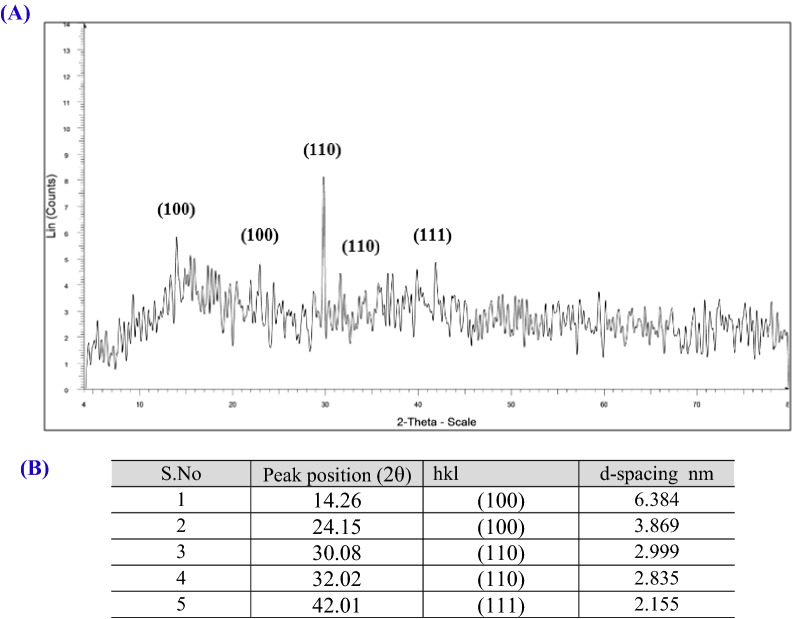
X-Ray Diffraction pattern of gelatin capped-silver nanoparticles bio synthesized by using Oscillatoria limnetica extract (**A** and **B**).

X-ray diffraction is currently a generic technique used to determine the crystallographic nature and atomic spacing of gelatin capped -AgNPs. Also it is an important characterization tool to confirm the formation of silver nanoparticles^70^. The XRD profile of the biosynthesized silver nanoparticles is shown in Fig. 5. Data of XRD revealed five well- defined diffraction peaks at 2θ values or Bragg reflections of 14.26°, 24.15°, 30.08°, 32.02° and 42.01° corresponding to (100), (100), (110), (110) and (111) planes, respectively, which were in agreement with the face-centered cubic (fcc) lattice structure or crystalline metallic silver (JCPDS no. 04-0783). In addition, other small intense peaks recorded at 39.81° and 59.62° attributed to planes (111) and (211), respectively. Furthermore, broad diffraction peak recorded between 15° and 20° indicate the amorphous gelatin phase acting as the capping agent^57^. Therefore, it is obvious that presence of amorphous and several XRD peaks which could be assigned to the formation of nano crystalline OG-AgNPs^71–73^. Furthermore, the analyzed characteristic peaks of the biosynthesized OG-AgNPs show low crystallinity of OG-AgNPs.

### Phytochemical screening of *O. limnetica* extract

Table 1 illustrates the extremely existence of some secondary metabolites as flavonoids, quinines, saponins and terpenoids in the aqueous extract of *O. limnetica*, which assists in the bioreduction and formation of nanoparticles as demonstrated by Hamouda et al.^5^. In the same context, Vijayaraghavan et al*.*^74^ explained that presence of many natural functional constituents in algae like alkaloids, terpenoids, proteins and flavonoids which may be responsible for biofabriction of AgNPs and biotransformation of metal ion to metal nanoparticles.

**Table 1:** Phytochemical test of Oscillatoria limnetica extract.

Phytochemicals	Result
Quinines	+
Flavonoids	+
Saponins	+
Terpenoids	+

### Phycotoxic effect of OG-AgNPs

#### Growth rate inhibition of *Chlorella spp* treated with OG-AgNPs

**Figure 6:**
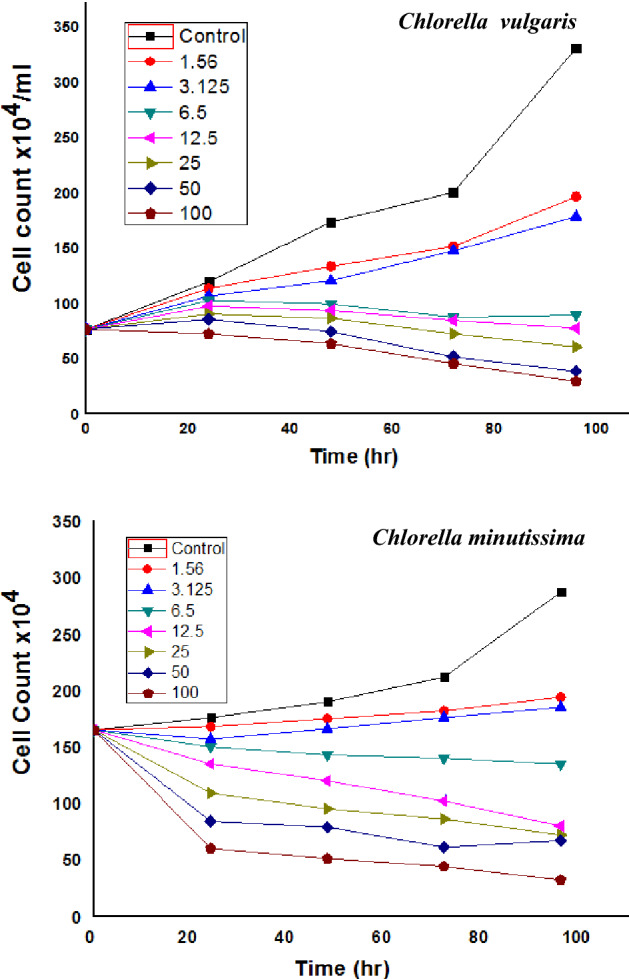
Effect of different concentrations of silver nanoparticles (OG-AgNPs) (100, 50, 25, 12.5, 6.5, 3.125, 1.5 and 0 μg/mL) on the growth of *Chlorella vulgaris* and *Chlorella minutissima* along the incubation period (24 h. interval) for 96 h (data are mean ± standard deviation).

**Figure 7:**
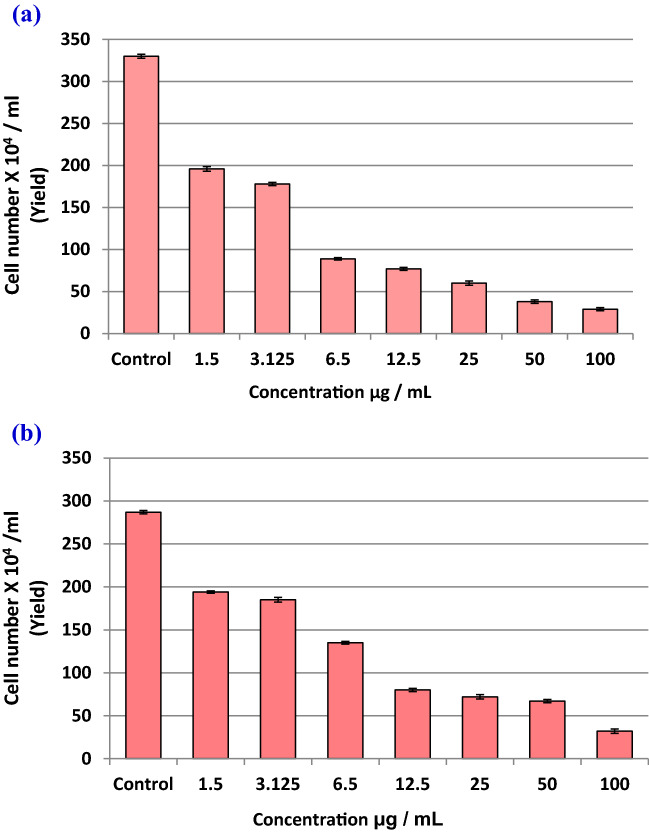
Yield of *Chlorella vulgaris* and *Chlorella minutissima* after 96 h exposure to different concentrations of OG-AgNPs (**a**) and (**b**), respectively.

**Figure 8:**
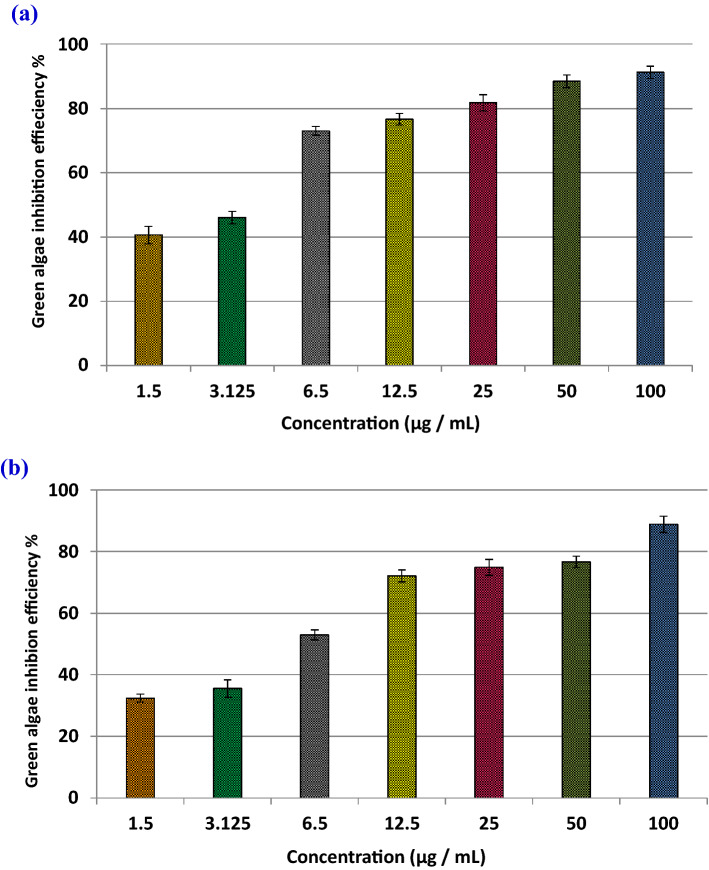
Green algae inhibition efficiency (%) (phycotoxicity) of OG-AgNPs on growth *Chlorella vulgaris* (**a**) and *Chlorella minutissima* (**b**), respectively. (data are mean ± standard deviation).

In this research, the response of the two freshwater microchlorophytes species of *Chlorella* to the toxic effect of OG-AgNPs was investigated via a set of growth analyses practice. The influence of various biosynthesized gelatin capped-AgNPs concentrations on growth of the two *Chlorella* species was illustrated in Figs. 6 and 7. Data indicated that exposure of *Chlorella spp*. to OG-AgNPs caused an evident change in cell counting between control and the different treatments. Furthermore, control cultures exhibit gradual increase in cell number (day 0: 76 cells ml^−1^ & 119 cells ml^−1^ for *C. vulgaris* and *C. minutissima*, respectively) and attained the highest value at the fourth day (day 4: cells ml^−1^ 330 & 287 cells ml^−1^ for *C. vulgaris* and *Chlorella sp*, respectively). Additionally, OG-AgNPs exposure induced reduction in cell number progressively with increasing nanoparticles concentration in dose dependent manner attending the following descending order 100 > 50 > 25 > 12.5 > 6.5 > 3.125 > 1.5 μg/ml. Thus, the maximum inhibition rate was detected at the fourth day with the highest concentration of OG-AgNPs (100 μg/mL), which reached 91.2% and 88.85% for *C. vulgaris* and *Chlorella sp*, respectively. Moreover, algae growth recorded 40.6% and 32.4% inhibition rate for *C. vulgaris* and *C. minutissima*, respectively in response to the lowest OG-AgNPs concentration (1.5 μg/mL) (Fig. 8).

**Figure 9:**
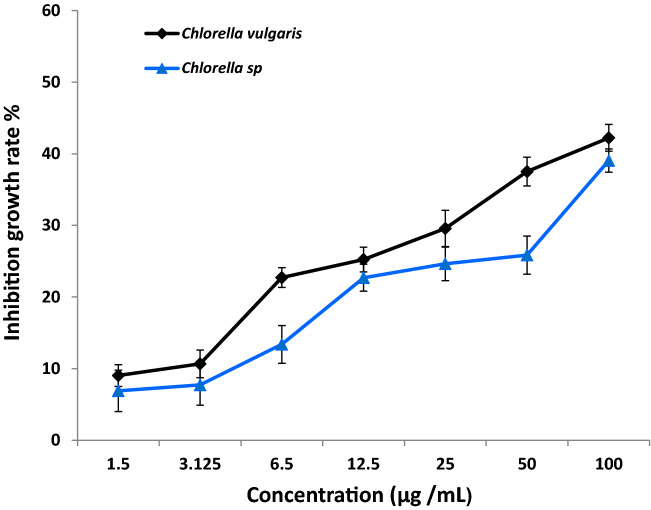
The percent inhibition of growth rate (average specific growth rate %) of *Chlorella vulgaris* and *Chlorella sp* after 96 h exposure to different concentrations of OG- AgNPs (100, 50, 25, 12.5, 6.5, 3.125, 1.5 and 0 μg/mL). Data taken from the mean of three replicates, and error bars signify the SD of these replicates.

The average specific growth rate exhibit the growth inhibitory effects of OG-AgNPs that attain dose dependent pattern that was drastically increased with rising concentrations^75,76^. The phycotoxic effect (IC_50_) was recognized at 3.705 μg/mL against *C. vulgaris* and 5.8 μg/mL for *Chlorella sp.* (Fig. 9). OG-AgNPs caused decline in cell viability for each of the investigated Chlorella species inducing a marked toxic response, progressively increased with increasing nanoparticles concentrations. Results revealed the sensitivity of *C. vulgaris* towards AgNPs treatments more than *C. minutissima*. The current results are in agreement with other studies that has been documented the ability of AgNPs to reduce growth of some fresh algal species such as cyanobacteria *Microcystis aeruginosa,* green algae as *Dunaliella salina*, *Chlorella vulgaris*^30,31,77^. Many studies has been documented the potent toxicity effects of AgNPs on several microalgae^76^. The mechanism of OG-AgNPs toxic effect on algal cell may be attributed to their direct contact with the cells surface leading to aggregation of the cells and discharge of silver cation‏ which resulted in the formation of reactive oxygen species (ROS) and lipids peroxidation damage^28^. Also, Perreault et al*.*^78^ ascribed the aggregation of the cells induced by AgNPs exposure to decrease algae cell obtainability to light, resulting in reduction in absorption of essential nutrients from the environment and consequence cell damage. The noticeable OG-AgNPs sensitivity of both *Chlorella* species may be due to their higher cell membrane permeability facilitating higher penetration of AgNPs into cells referring to penetration of OG-NPs to cell wall of the investigated microalgae^79^ demonstrating the algicidal effect of the prepared OG-AgNPs^80,81^.

#### Photosynthetic response of *Chlorella* spp. to OG-AgNPs treatment

**Figure 10:**
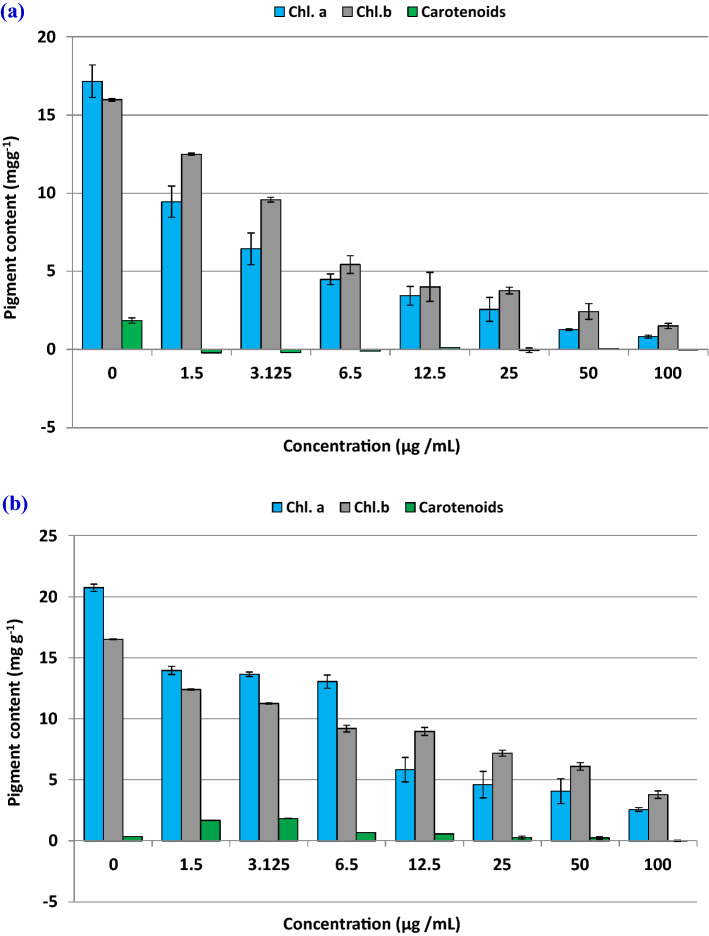
Decrease of photosynthetic pigments content (Chl. a ,Chl.b and carotenoids) in *Chlorella vulgaris* (**a**) and *Chlorella minutissima* (**b**) micro green algae exposed to gelatin-capped silver nanoparticles for 96 h.The experiments were performed in triplicate and results are represented as the mean with standard deviations.

The photosynthetic pigments content was assessed for additional investigation of the toxic effect of OG-AgNPs on *Chlorella* spp*.* Photosynthetic pigments especially chlorophyll a are considered an effectual parameter for algal growth. Distinct reduction in photosynthetic pigments content (chlorophyll a, chlorophyll b & carotenoids) relative to control (without AgNPs) was illustrated in Fig. 10. This is decrement in photosynthetic pigment contents signifying that gelatin capped-silver nanoparticles are able to influence pigmentation obviously as a consequence of growth inhibition, particularly at high concentrations of OG-AgNPs.

#### Sensing property of OG-AgNPs towards hydrogen peroxide

**Figure 11:**
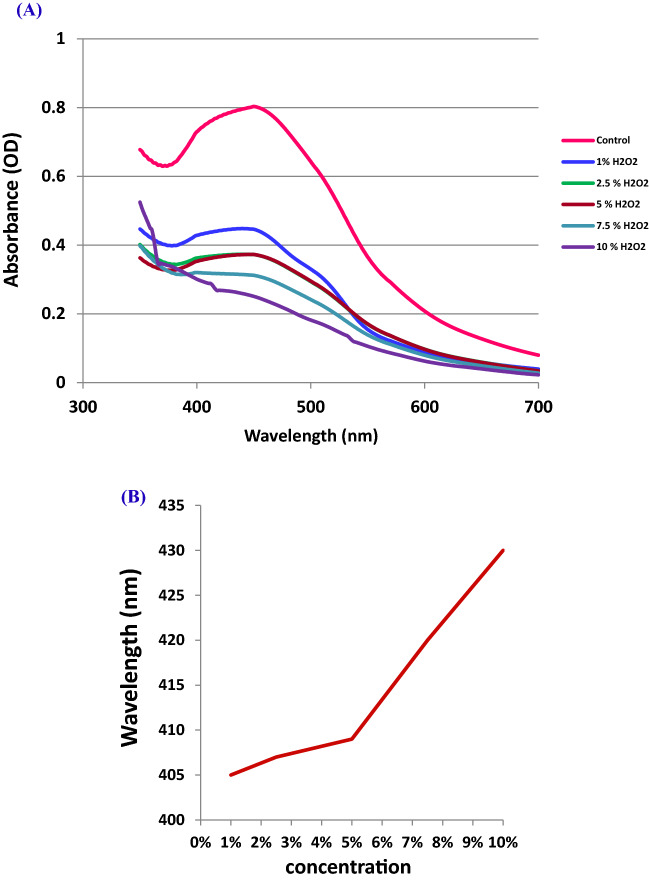
Change in SPR peak position after the introduction of H_2_O_2_ solution with different concentration to OG-AgNPs solution at a volume ratio 1:1.5 (**A**) and (**B**) figure of H_2_O_2_ concentration against the SPR peak position.

Reactive oxygen specie (ROS) such as hydrogen peroxide has been described as a potential contaminant in biological systems and aquatic environment. It is a product of oxygen metabolic reactions in biological systems. Once H_2_O_2_ is found in a living system in excessive, it participates in DNA damage^33,43^. So, the determination of H_2_O_2_ concentration, particularly at trace ppm level is of great importance for applying in numerous domains such as medical and industrial processes. The use of Ag-nanocomposite as colorimetric sensors for the detection of H_2_O_2_ has been approved to be auspicious method^20,82^. In this study, sensing character of the biogenic gelatin capped Ag-NPs formed via a completely green approach were investigated. Silver nanoparticles are able to reduce hydrogen peroxide with silver oxidation. Therefore, utilizing this reaction mechanism between hydrogen peroxide and silver nanoparticles for colorimetric detection of H_2_O_2_ could be accomplished simply. Figure 11A illustrated the change in the maximum surface plasmon resonance (SPR) peak position of the OG-AgNPs due to introducing H_2_O_2_ at various concentrations. The change in optical characteristics was observed 2 min only after introducing H_2_O_2_ solution in the gelatin coated silver nanoparticles preparation. Data illustrated a red-shift in the SPR peak position of OG-AgNPs from 405 to 430 nm by increasing the concentration of H_2_O_2_ solution which could be interpreted according to the catalytic reduction reaction between silver and hydrogen peroxide that leads to H_2_O_2_ decomposition^83^ In addition, the shifting in SPR band may be induced by some aggregation of nanoparticles and an increase in the particle size correlated with a destruction of the gelatin shell that coating AgNPs which stabilizing the nanoparticles and declining the distance between the particles (Fig. 11A,B). Once the concentration of the hydrogen peroxide solution increased, a decline in the maximum intensity (ʎ_max_) was detected. This remarked decline in the absorbance intensity indicates decrease in the Ag-NPs concentration. Consequently, the elevated peroxide concentration, the higher decline in the Ag-NPs concentration and therefore, the Ag-NPs SPR peak intensity was also decreased distinctively. In this study, the nanoparticles were capped by gelatin layer and results demonstrated catalytic reaction between silver and hydrogen peroxide proceeds through the protection layer of gelatin around the Ag-NPs as the addition of H_2_O_2_ in the solution induces the degradation of the gelatin protected Ag-NPs with oxidation of Ag to Ag^+^ ions by production of reactive radical species. Subsequently, the SPR absorbance was also decreased. The reactive radical species initiates the degradation of gelatin protected AgNPs. This will increase particle aggregation and decrease the amount of smaller AgNPs concentration and therefore, decreasing of AgNPs SPR peak intensity.

**Figure 12:**
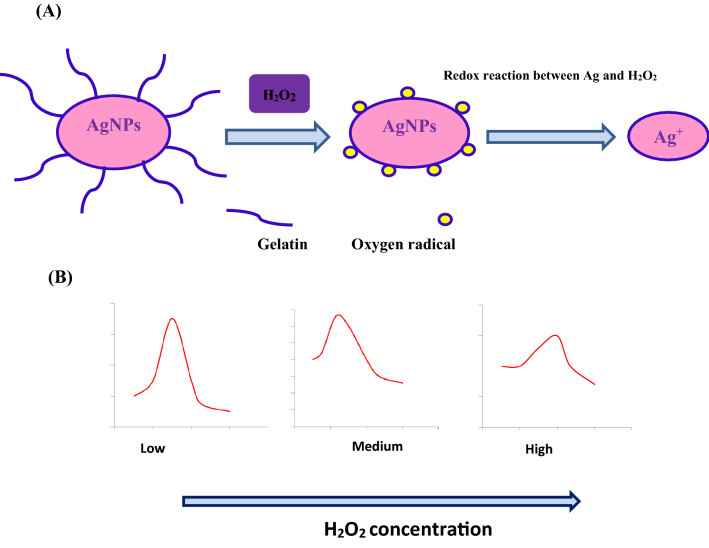
A schematic model of the probable mechanism of a catalytic reaction between OG-AgNPs and H_2_O_2_ (**A**) and a decrease in the absorbance intensity with increasing in H_2_O_2_ concentration (**B**).

Furthermore, the color of the solutions altered gradually from dark brown to colorless after the addition of H_2_O_2_, depending on hydrogen peroxide concentration (1–10% H_2_O_2_) confirming H_2_O_2_ sensing^84^. A schematic illustration of the probable mechanism is provided in Fig. 12A,B.

## Conclusion

In conclusion, very stable, small and water soluble gelatin coated AgNPs (OG-AgNPs) were successively synthesized with high biocompatibility by completely green approach. The synthesis process was attained using aqueous extract of *O. limnetica* fresh biomass and gelatin that act as a reducing and capping agent respectively while AgNO_3_ was used as the silver precursor without using of any accelerator. Additionally, OG-AgNPs exhibited high toxicity on the cellular viability and photosynthesis of *C. vulgaris* and *C. minutissima.* From these results the toxicological response of *Chlorella species* to OG-AgNPs shows a substantial increase with increasing time and concentration. Finally, it is necessary to protect aquatic organisms from AgNPs exposures as algae populations that are implicated in the environmental preserving the oxygen production in addition to the food chain and the variations might have unsafe results on the whole functioning of the aquatic ecosystem. The biosynthesized OG-AgNPs performed as good sensor to one of the reactive oxygen species (H_2_O_2_). The current research presents a very sensitive, facile and rapid method for H_2_O_2_ detection (within 2 min). Furthermore, this method poses an auspicious non-skill dependent technique with high sensitivity for detection of H_2_O_2_ in food, medical, industrial analysis and remediation or controlling the environmental contaminant. Hence, this method may open up novel perception for the expansion of colorimetric sensors depends on the specific catalytic reactivity of OG-AgNPs.

## Data Availability

The datasets spent and/or analyzed during this study are available from the corresponding author on reasonable request.
